# Association between dietary inflammatory potential and risk of developing gestational diabetes: a prospective cohort study

**DOI:** 10.1186/s12937-021-00705-5

**Published:** 2021-06-02

**Authors:** Sanaz Soltani, Azadeh Aminianfar, Hossein Hajianfar, Leila Azadbakht, Zahra Shahshahan, Ahmad Esmaillzadeh

**Affiliations:** 1 Department of Community Nutrition, School of Nutritional Sciences and Dietetics, Tehran University of Medical Sciences, P.O. Box 14155-6117, Tehran, Iran; 2 Research Center for Biochemistry and Nutrition in Metabolic Diseases, Kashan University of Medical Sciences, Kashan, Iran; 3 Food Security Research Center, Isfahan University of Medical Sciences, Isfahan, Iran; 4 Food Safety Research Center (salt), Semnan University of Medical Sciences, Semnan, Iran; 5 Department of Community Nutrition, Isfahan University of Medical Sciences, Isfahan, Iran; 6 Diabetes Research Center, Endocrinology and Metabolism Clinical Sciences Institute, Tehran University of Medical Sciences, Tehran, Iran; 7 Department of Gynecology, School of Medicine Science, Isfahan University of Medical Sciences, Isfahan, Iran; 8 Obesity and Eating Habits Research Center, Endocrinology and Metabolism Molecular -Cellular Sciences Institute, Tehran University of Medical Sciences, Tehran, Iran

**Keywords:** Cohort, Dietary inflammatory potential, Gestational diabetes mellitus, Pregnancy

## Abstract

**Background:**

Limited and inconsistent data are available regarding the relationship between the dietary inflammatory potential (DIP) and risk of gestational diabetes mellitus (GDM).

**Objective:**

The present prospective study aimed to evaluate the association between DIP score during the first trimester of pregnancy and risk of developing GDM among Iranian women.

**Methods:**

In this prospective cohort study, 812 pregnant women aged 20–40 years, who were in their first trimester, were recruited and followed up until week 24–28 of gestation. Dietary intakes of study subjects were examined using an interviewer-administered validated 117-item semi-quantitative food frequency questionnaire (FFQ). DIP score was calculated from 29 available food parameters based on earlier literature. The results of a fasting plasma glucose concentration and a 50-g, 1-h oral glucose tolerance test, between the 24th and 28th week of gestation, were used to diagnose GDM. The risk of developing GDM across quartiles of DIP score was estimated using Cox regression in several models.

**Results:**

At study baseline, mean (SD) age and BMI of study participants were 29.4 (±4.84) y and 25.14 (±4.08) kg/m^2^, respectively. No significant association was found between DIP score and risk of GDM in the crude model (RR: 1.01; 95% CIs: 0.71–1.45). When we adjusted for age the association did not alter (RR: 1.04; 95% CIs: 0.72–1.48). Even after further adjustment for maternal weight gain we failed to find a significant association between DIP score and risk of GDM (RR: 0.97; 95% CIs: 0.66–1.41).

**Conclusion:**

We found no significant association between DIP and risk of developing GDM. Further longitudinal studies among other populations are needed to elucidate the association between DIP score and GDM.

**Supplementary Information:**

The online version contains supplementary material available at 10.1186/s12937-021-00705-5.

## Introduction

Gestational diabetes mellitus (GDM) is a condition of glucose intolerance first diagnosed in the second or third trimester of pregnancy. It is associated with an increased risk of current and future health problems in mothers (such as preeclampsia (PE), cesarean delivery, metabolic syndrome, type 2 diabetes mellitus (T2DM) and cardio vascular disease (CVD)) and their offspring (such as macrosomia, hyperbilirubinemia, respiratory distress syndrome, T2DM, subsequent obesity, metabolic syndrome, impacted neurodevelopmental outcome and neuropsychiatric morbidity) [1–11]. The prevalence of GDM varies across countries depending on population characteristics [12, 13] and different diagnostic criteria used [14–16]. In the Middle East and some North African countries, approximately 13% of all pregnant women are diagnosed with GDM [17].

Although the etiology of GDM remains poorly known, several risk factors including age, overweight or obesity, ethnicity, family history of diabetes, and history of GDM have been proposed [18–21]. In addition, earlier studies have suggested GDM as a pro-inflammatory state in which serum levels of pro-inflammatory cytokines are elevated [22]. Diet has been linked both to GDM [23] and inflammation [24]. The association of dietary intakes with GDM might be explained by its pro-inflammatory properties. To capture the overall inflammatory potential of a diet, Shivappa et al. have developed an index based on dietary inflammatory potential (DIP) according to the literature [25]. This index estimates the diet’s inflammatory potential in a continuous scale from anti-inflammatory to pro-inflammatory range. It has been shown to predict elevated inflammatory biomarkers [26–28]. Earlier studies have linked DIP score to several metabolic condition including cardiovascular disease [29], obesity [30], metabolic syndrome (MetS) [31, 32] and diabetes [33]. We are aware of only two studies that have investigated the association between DIP and GDM [34, 35]. Sen et al. in a prospective, cohort study on 1808 women in the United States found that adherence to a pro-inflammatory diet was associated with a lower odds of GDM [34]. In contrast, a case-control study in Iranian women showed that a pro-inflammatory diet was associated with increased odds of GDM [35]. It must be kept in mind that most reports on diet-disease associations came from western countries and limited data exist in this regard from Middle Eastern countries. Studying the association between DIP score and GDM is particularly relevant for Middle Eastern countries, where dietary intake and consequently dietary inflammatory potential is very different from that found in other parts of the world. Given the limited and inconsistent findings about the association between the inflammatory potential of diet and risk of GDM, the aim of this study was to evaluate the association between DIP score during first trimester of pregnancy and risk of GDM among Iranian women.

## Methods

### Study design and participants

**Fig. 1:**
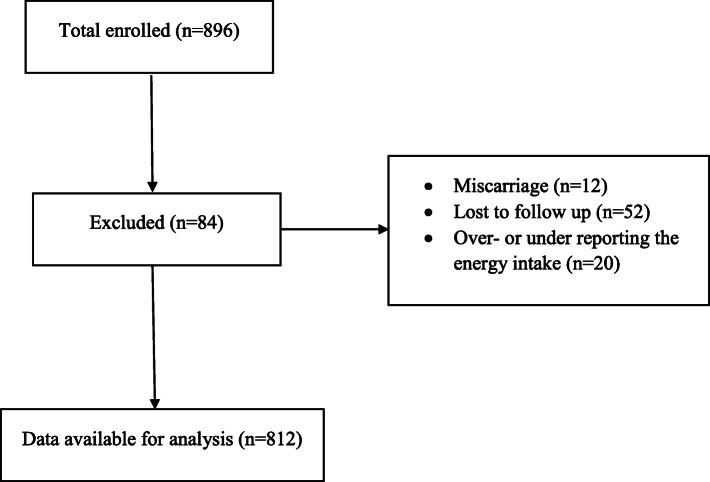
Flow chart of study population

The current prospective observational study was conducted among Isfahanian pregnant women who attended at health centers during 2015–2016. A sample of 896 pregnant women aged 20–40 was selected from 20 various health centers by multistage cluster random sampling method. Women who were singleton pregnant, at their first trimester, without any medical condition and medication use were eligible to participate in the study. Women who were smokers or had twin pregnancies were not included. As misreporting of energy intake might affect the estimates of nutrient intakes, which in turn would lead to misclassification of study participants [36], we preferred to exclude individuals with a total energy intake outside the range of 800–4200 kcal/day (*n* = 20). We also excluded women who experienced miscarriage (*n* = 12), or who did not complete the study (*n* = 52) from the present analysis (Fig. 1). After these exclusions, a total of 812 women remained for the analysis. Informed written consent was obtained from all study participants. This study was approved by the Research Council of School of Nutritional Sciences and Dietetics, Tehran University of Medical Sciences, Tehran, Iran (Ethics code: IR.TUMS.VCR.REC.1398.382).

#### Assessment of dietary intakes

Usual dietary intakes of study participants were examined using an interviewer-administered validated 117-item semi-quantitative food frequency questionnaire (FFQ) [37] at their first visit between week 8 and 16 of pregnancy. All women were requested to report their frequency consumption of listed food items in the FFQ, based on commonly used units or portion sizes, from the beginning of their pregnancy. The frequency response categories were nine multiple choice categories varying from “never or less than once a month” to “6 or more times per day” depending on the nature of food. Because the Iranian food composition table is incomplete, we used the United States Department of Agriculture (USDA) food composition table to analyze foods and beverages; however, in the dataset of USDA in the software, we modified some traditional foods and beverages based on the Iranian food composition table, which were not listed in the USDA food composition table. This means that for almost 98% of foods we had in the FFQ, we used the USDA database. For some food items that were not available there (for example Iranian local breads like Lavash and Barbari) and we had the nutrient composition of these foods in Iranian food composition table [38], we added these foods to the database of the software. Nutrient intakes for each participant was calculated using the USDA food composition database that was modified for Iranian foods.

The validity and reliability of this FFQ had been previously evaluated [37]. The validity and reliability of FFQ was assessed using the 24-h dietary recalls (two 24-h recalls per month) as gold standard. Based on findings of validation study, the questionnaire seems to provide reasonably valid measures of long-term dietary intakes. The correlation coefficients for dietary intakes obtained from the FFQ and those from the average of multiple 24-h dietary recalls were 0.65 for dietary fats, 0.75 for saturated fatty acids (SFAs) and 0.82 for dietary cholesterol. The corresponding values for vitamin E, vitamin C and β-carotene were 0.49, 0.65 and 0.68, respectively. Correlation coefficients for the reliability of the FFQ were 0.72 for dietary fats, 0.79 for dietary SFAs and 0.87 for dietary cholesterol [37].

#### Construction of DIP score

FFQ-derived dietary data was used to calculate DIP score for all participants. For constructing this score, we used Shivappa et al.’s method [25]. They found that a total of 45 specific foods and nutrients were associated with one or more of the inflammatory [Interleukin-1β (IL-1β), Interleukin-6 (IL-6), Tumor Necrosis Factor-α (TNF-α) or CRP] or anti-inflammatory biomarkers [Interleukin-4 (IL-4) and Interleukin-10 (IL-10)]. Then, they scored the inflammatory potential for each food parameter according to whether it increased inflammatory or decreased anti-inflammatory factors (+ 1), or it decreased inflammatory or increased anti-inflammatory factors (− 1), or had no effect (0) on inflammatory or anti-inflammatory biomarkers. They calculated world mean and standard deviation for each of the 45 food parameters based on 11 data sets from 11 countries in different parts of the world. In the present study, due to lack of access to some food items’ information (including alcohol, eugenol, ginger, n-3 fatty acids, n-6 fatty acids, trans fat, saffron, turmeric, flavan-3-ol, flavones, flavonols, flavonones, anthocyanidins, isoflavones, thyme/oregano and rosemary), 29 food parameters were used in computing DIP score: including 8 pro-inflammatory parameters (energy, carbohydrate, fat, protein, cholesterol, saturated fat, vitamin B12 and iron) and 21 anti-inflammatory parameters (mono-unsaturated fatty acids (MUFAs), poly unsaturated fatty acids (PUFAs), fiber, vitamin B6, folic acid, niacin, riboflavin, thiamin, vitamin A, vitamin C, vitamin D, vitamin E, b-carotene, caffeine, pepper, onion, garlic, tea, zinc, selenium, and magnesium). In order to reduce between-subject variation, we calculated the energy-adjusted values of these food parameters using residual method [39]. Then, for each participant and each food parameter, a z score was computed by subtracting the “standard global mean” from the amount consumed by each subject and dividing this value by the “global standard deviation”. Global means and standard deviations were obtained from the study of Shivappa et al. [25]. This derived z score was then converted to a centered percentile score to reduce skewness. Subsequently, we multiplied this score by the respective food parameter effect score derived from the study of Shivappa et al. [25]. Finally, the total DIP score was computed by summing up all 29 foods’ DIP score.

#### Assessment of GDM

Pregnant women, not previously diagnosed with diabetes, were screened for GDM at 24–28 weeks of gestation with a 50-g oral glucose tolerance test (OGTT) and plasma glucose measurement when participant is fasting (for at least 12 h) and at 1 h. Plasma glucose levels were measured with glucose oxidase method using commercially available reagents (Bio System, Tehran, Iran) adapted to a Selecta auto analyzer. Women were diagnosed with GDM if fasting plasma glucose concentration was more than 95 mg/dl and/or 1-h post-challenge plasma glucose concentration was ≥140 mg/dl [40, 41].

#### Assessment of other variables

Required information on other variables including age, number of family, occupation status, educational level, number of previous pregnancies and history of intrauterine growth retardation (IUGR), stillbirth, abortion, preterm delivery and cesarean section was obtained from a pre-tested questionnaire at study baseline. We asked some members of the research team and some study population, to examine content and face validity of the questionnaires, prior to study beginning. Physical activity of study participants was assessed using the General Practice Physical Activity Questionnaire (GPPAQ) [42]. Body weight was quantified at study baseline using a balanced digital scale to the nearest 100 g, in light clothing and barefoot. This was also done again at the second visit when the participants came to the laboratory to collect blood samples. Height was measured with a tape measure while the subjects were in a standing position without shoes. Body mass index (BMI) was calculated through the division of weight in kilograms by height in meters squared. Weight gain was computed as the difference in weight at the second visit (24–28 weeks of pregnancy) minus weight of participant at study baseline.

### Statistical analysis

First, we categorized participants into quartiles of DIP score. Then, general characteristics of participants were compared across quartiles of DIP score using one-way ANOVA for continuous variables and chi-square test for categorical variables. To compare age- and energy-adjusted intakes of nutrients across quartiles of DIP score, we used analysis of covariance (ANCOVA) with Bonferroni correction. To estimate relative risks (RRs) and 95% confidence intervals (CIs) for GDM across categories of DIP score, we used Cox regression in several models. In the first model, we did adjustment for age, as a continuous variable. Then we additionally adjusted for physical activity (inactive/moderately inactive/moderately active/active), education (high school graduate/university graduate), occupational status (housewife/employee), family number (continuous), history of abortion (yes/no), history of stillbirth (yes/no), history of preterm delivery (yes/no), history of cesarean section (yes/no), number of previous pregnancies (≤2/≥3) and baseline-BMI (continuous) in the second model. Finally, we added maternal weight gain (as continuous) into the model. All confounders were chosen based on previous publications [20, 34, 43, 44]. In these analyses, the lowest quartile of DIP score was taken as the reference category. To calculate the trend of RRs across increasing categories of DIP score, we considered the categories as ordinal variable. The risk of GDM for each unit increase in continuous DIP score was also computed using Cox regression. We used SPSS software (SPSS Inc., version 22) for all statistical analyses. *P* value < 0.05 was considered statistically significant.

## Results

At study baseline, mean (SD) age and BMI of study participants were 29.4 (±4.84) y and 25.14 (±4.08) kg/m^2^, respectively. The overall mean DIP in the study participants was − 0.0028 (SD: ±1.72). We documented 231 incident GDM pregnancies. General characteristics of study participants across quartiles of DIP scores are provided in Table 1. Women with a higher overall DIP score were more likely to have a history of stillbirth (*P* = 0.03) and less likely to have higher BMI at study baseline (*P* = 0.02). No other significant difference was observed in terms of other general characteristics across quartiles of DIP score among women.

**Table 1:** General characteristics of study participants across quartiles of dietary inflammatory potential (*n* = 812)^a^

	Quartiles of the dietary inflammatory index				P^b^
	Q1 (<−1.24)	Q2 (− 1.24 to + 0.04)	Q3 (+ 0.04 to + 1.19)	Q4 (> + 1.19)	
Age (years)	29.57 ± 4.71	29.77 ± 4.73	29.33 ± 4.82	28.95 ± 5.13	0.38
Baseline-BMI (kg/m2)	25.81 ± 4.25	25.16 ± 3.99	24.95 ± 3.94	24.61 ± 3.99	0.02
Maternal weight gain^c^ (kg)	7.49 ± 2.38	7.75 ± 3.00	8.00 ± 3.30	7.58 ± 2.19	0.28
Family number	2.72 ± 0.78	2.72 ± 0.75	2.70 ± 0.87	2.69 ± 0.78	0.98
GDM (yes) (%)	30.6	29.4	24.9	31.1	0.50
University graduated (%)	52.6	52.3	56.9	55.6	0.74
Employed (%)	23.7	17.4	20.3	17.5	0.35
Physically active (%)	8.2	11.2	7.7	5.8	0.34
Number of previous pregnancies (≥3) (%)	18.4	22.8	16.8	18.4	0.45
History of IUGR (yes) (%)	5.1	5.1	3.1	5.2	0.69
History of abortion (yes) (%)	22.4	24.4	15.2	18.9	0.11
History of cesarean section (yes) (%)	80.9	77.9	81.5	78.4	0.75
History of stillbirth (yes) (%)	0.5	0.0	0.5	2.6	0.03
History of preterm delivery (yes) (%)	2.6	0.5	1.5	3.6	0.16

*GDM* Gestational diabetes mellitus, *IUGR* Intrauterine growth retardation

^a^All values are mean ± SD unless indicated

^b^*P*-values were obtained from ANOVA or χ2 test, where appropriate

^c^Increment in the weight from the study baseline to the second visit at weeks 24–28 of pregnancy

Age- and energy-adjusted dietary intakes of study participants across quartiles of DIP scores are presented in Table 2. Women with the greatest DIP score consumed higher energy, fat, MUFA and saturated fat but lower carbohydrate, protein, dietary fiber, PUFA, vitamins B6, folic acid, A, C and E, β-carotene, magnesium and iron than those with the lowest score (all *P*-values were <  0.001). Dietary intakes of other nutrients were not significantly different across quartiles of DIP score.

**Table 2:** Dietary intakes of study participants across quartiles of dietary inflammatory potential (*n* = 812)^*^

	Quartiles of the dietary inflammatory potential				P^**^
	Q1 (<− 1.24)	Q2 (− 1.24 to + 0.04)	Q3 (+ 0.04 to + 1.19)	Q4 (> + 1.19)	
Nutrients					
Energy (kcal/d)	1973.38 ± 43.72	1820.15 ± 43.64	1985.44 ± 43.61	2299.87 ± 43.76	< 0.001
Carbohydrate (g/d)	287.40 ± 2.27	280.84 ± 2.29	266.07 ± 2.26	253.12 ± 2.33	< 0.001
Fat (g/d)	65.73 ± 0.94	69.38 ± 0.95	74.62 ± 0.94	82.85 ± 0.97	< 0.001
Protein (g/d)	90.39 ± 1.01	85.28 ± 1.02	85.66 ± 1.01	76.93 ± 1.04	< 0.001
Fiber (g/d)	37.02 ± 0.45	32.85 ± 0.46	30.13 ± 0.45	24.77 ± 0.46	< 0.001
Cholesterol (mg/d)	244.18 ± 6.08	234.79 ± 6.14	251.77 ± 6.06	239.00 ± 6.24	0.22
MUFA (g/d)	19.28 ± 0.30	19.65 ± 0.30	20.98 ± 0.30	20.55 ± 0.31	< 0.001
PUFA (g/d)	13.76 ± 0.30	13.75 ± 0.31	14.20 ± 0.30	12.37 ± 0.31	< 0.001
Saturated fat (g/d)	21.22 ± 0.46	23.12 ± 0.47	25.41 ± 0.46	26.86 ± 0.48	< 0.001
Vitamin B12 (μg/d)	4.69 ± 0.11	4.61 ± 0.11	4.86 ± 0.11	4.90 ± 0.12	0.26
Vitamin B6 (mg/d)	2.15 ± 0.02	1.91 ± 0.02	1.81 ± 0.02	1.67 ± 0.02	< 0.001
Folic acid (μg/d)	583.23 ± 9.19	530.35 ± 9.29	485.51 ± 9.16	413.30 ± 9.43	< 0.001
Vitamin A (RE/d)	950.22 ± 19.11	726.54 ± 19.32	625.11 ± 19.06	516.33 ± 19.61	< 0.001
Vitamin C (mg/d)	279.16 ± 6.46	241.56 ± 6.53	199.31 ± 6.45	167.39 ± 6.63	< 0.001
Vitamin D (μg/d)	2.73 ± 0.13	2.86 ± 0.13	2.97 ± 0.13	2.60 ± 0.13	0.26
Vitamin E (mg/d)	11.48 ± 0.21	10.26 ± 0.21	9.71 ± 0.21	8.75 ± 0.21	< 0.001
β-Carotene (μg/d)	8199.75 ± 188.02	5277.76 ± 190.00	3884.03 ± 187.46	2779.14 ± 192.95	< 0.001
Caffeine (g/d)	72.32 ± 3.95	63.54 ± 3.99	63.83 ± 3.94	64.40 ± 4.05	0.33
Mg (mg/d)	467.05 ± 3.75	414.71 ± 3.79	391.90 ± 3.74	344.73 ± 3.85	< 0.001
Ca (mg/d)	1351.38 ± 29.14	1292.33 ± 29.45	1298.06 ± 29.06	1312.75 ± 29.91	0.47
Fe (mg/d)	15.57 ± 0.16	13.66 ± 0.16	12.75 ± 0.16	11.04 ± 0.17	< 0.001

^*^All values are mean ± SE

^**^All values were adjusted for age and energy, except for dietary energy intake, which was only adjusted for age using ANCOVA

Table 3 indicates the multivariable-adjusted ratios for GDM across quartiles of DIP scores. We found no significant association between DIP score and risk of GDM in crude model (RR: 1.01; 95% CIs: 0.71–1.45). When we adjusted for age, the association did not alter (RR: 1.04; 95% CIs: 0.72–1.48). Even after further adjustment for maternal weight gain, we failed to find a significant association between DIP score and risk of GDM (RR: 0.97; 95% CIs: 0.66–1.41).

**Table 3:** Multivariable-adjusted ratios for GDM across quartiles of dietary inflammatory potential (*n* = 812)^a^. (Odds ratios and 95% confidence intervals)

	Quartiles of the dietary inflammatory potential							Each unit increment in DIP		P_trend_
	Q1 (<−1.24)	Q2 (− 1.24 to + 0.04)		Q3 (+ 0.04 to + 1.19)		Q4 (> + 1.19)				
	RR	RR	95% CI	RR	95% CI	RR	95% CI	RR	95% CI	
Crude	1.00	0.96	0.67–1.38	0.81	0.55–1.18	1.01	0.71–1.45	0.98	0.91–1.05	0.85
Model 1	1.00	0.95	0.66–1.36	0.82	0.56–1.19	1.04	0.72–1.48	0.98	0.91–1.06	0.98
Model 2	1.00	0.93	0.64–1.35	0.80	0.54–1.18	0.97	0.66–1.41	0.97	0.89–1.05	0.69
Model 3	1.00	0.93	0.64–1.34	0.79	0.54–1.17	0.97	0.66–1.41	0.97	0.89–1.05	0.68

*GDM* Gestational diabetes

^a^The analysis of Cox regression was used to determine the RR and 95% confidence interval

Model 1: Adjusted for age (continuous)

Model 2: Additional adjustment were made for physical activity (inactive/moderately inactive/moderately active/active), education (high school graduate/university graduate), occupation status (housewife/employee), family number (continuous), history of stillbirth (yes/no), history of preterm delivery (yes/no), history of cesarean (yes/no), history of abortion (yes/no), pregnancy number (≤2/≥3) and baseline-BMI (continuous)

Model 3: Additional adjustment were made for maternal weight gain (continuous)

## Discussion

In this prospective cohort study, we examined the association between DIP score and risk of GDM. We found no significant association between DIP score and risk of GDM. To the best of our knowledge, this is the first prospective cohort study in the Middle-East assessing DIP score in relation to the incidence of GDM.

GDM is associated with increased risk of maternal and fetal health complications. In the current study, in order to evaluate the association between a pro-inflammatory diet and risk of GDM, we used a previously derived score of DIP, which has been previously validated with several inflammatory markers, including CRP, TNF-a, and IL-6 [26, 27, 45, 46]. In addition to the association with inflammatory markers, the DIP score has also been linked with glycaemia and risk of insulin resistance [33, 47, 48]. Furthermore, several prior studies have provided evidence on adverse effects of higher DIP on risk of developing prediabetes and type 2 diabetes [33, 48]. To date, very limited attention has been paid to the role of DIP in GDM. To our knowledge, only two previous studies examined the association between DIP and risk of GDM [34, 35]. In a prospective cohort study on 1808 mother-child pairs in Massachusetts, Sen et al. [34], found a significant inverse association between pro-inflammatory diet and risk of GDM in all participants, particularly in overweight women. However, they did not observe any significant association between DIP and rates of isolated hyperglycemia or impaired glucose tolerance. It should be noted that Sen et al. used mean dietary intakes of mothers that were assessed in the first- and second-trimester to compute DIP; however, in the present study, we used only dietary intakes in the first trimester of pregnancy. The discrepant results may also be explained by different study population, sample size as well as adjusting for different covariates. Shivappa et al. [35] in a hospital-based case-control study among Iranian women reported that individuals with more pro-inflammatory diets were at greater odds of GDM compared to those with more anti-inflammatory diets. The differences in findings likely resulted from differences in study design. In addition, although they used the similar method to construct the DIP score, there are some differences in the components used for DIP calculation. Shivappa et al. reported that 32 food parameters from the FFQ were used to calculate DIP, however, they applied 31 food parameters for DIP construction (including energy, carbohydrate, protein, total fat, fiber, cholesterol, saturated fat, mono-unsaturated fat, poly unsaturated fat, omega-3, omega-6, trans fat, niacin, thiamin, riboflavin, vitamin B12, vitamin B6, iron, magnesium, selenium, zinc, vitamin A, vitamin C, vitamin D, vitamin E, folic acid, beta carotene, garlic, turmeric, onion, caffeine). In the current study, we have used 29 food parameters from the FFQ and we did not have information about omega-3, omega-6, trans fat and turmeric. However, the information of pepper and tea was available in our study. Regarding the paucity of knowledge on the association of DIP and GDM, further prospective studies are warranted in this field.

We failed to find any significant association between DIP score and risk of GDM. However, DIP might be associated with risk of GDM through the effect of a pro-inflammatory diet on insulin resistance by increasing the inflammatory mediators. Recent evidence suggests a positive relationship between inflammatory markers including IL-1β, IL-6, CRP and TNF-α and insulin resistance [49]. TNF-α, one of the most commonly studied cytokines in relation to insulin resistance, has been shown to inhibit insulin receptor signaling. Moreover, TNF-α with more lipolytic and less liposynthetic activities [50], induces an increase in circulating free fatty acids (FFA), which in turn leads to insulin resistance [51].

The present study had several strengths. First, this is the first prospective cohort study in the Middle East that examined the association of DIP score and risk of GDM. Second, many potential confounders were included in the multivariate analysis to reach an independent association between DIP and risk of GDM. Third, the DIP score was estimated using a validated method. However, some limitations should also be addressed. The FFQ we applied had been validated among non-pregnant population. There is no pregnancy-specific FFQ in Iran. However, this questionnaire has been used in other studies among pregnant women in the country and it seems that it provides valid measures of dietary intakes during pregnancy due to predicting several pregnancy-related outcomes [52, 53]. In addition, as with all epidemiologic studies that used FFQ, misclassification of study participants due to measurement errors cannot be entirely excluded. Moreover, as mentioned, we did not have information about 16 food parameters in calculating DIP score (including alcohol, eugenol, ginger, n-3 fatty acids, n-6 fatty acids, trans fat, saffron, turmeric, flavan-3-ol, flavones, flavonols, flavonones, anthocyanidins, isoflavones, thyme/oregano and rosemary). Almost all these dietary parameters have anti-inflammatory properties which may cause our final DIP score to reflect inflammatory potential of the diet rather than its anti-inflammatory state compared with other studies. In addition, despite controlling for several confounders, residual confounding cannot be eliminated. In the current study, we estimated the DIP score based on a single measurement of dietary intakes prior to GDM diagnosis, during the first trimester of pregnancy; however, it may not accurately represent an individual’s DIP score. Therefore, the repeated measurement of dietary intakes since the last menstrual period could provide a better assessment of DIP prior to predict GDM. Moreover, no information was collected about history of gestational diabetes in the current study. The present DIP score was based on previous studies. Although the application of this index to predict circulating inflammation in Iranian pregnant women has earlier been validated [53], we did not further examine the validity of this index in the current study population. Nevertheless, as this score has been used to predict different inflammatory-related conditions in the country, it seems that this index works well in our population. Finally, the generalizability of our findings may be limited due to the study participants’ demographic characteristics.

## Conclusion

In conclusion, we found no significant association between DIP and risk of developing GDM. Further longitudinal studies among other populations are needed to elucidate the association between DIP score and GDM.

## Supplementary Information

**Additional file 1: Supplementary Table 1.** Sample of the questionnaire. For each food item a portion size was considered. The frequency response options ranged from never to more than 6 times a day for each row.

## Data Availability

The data are not publicly available.

## Abbreviations

DIP: Dietary Inflammatory Potential
GDM: Gestational Diabetes Mellitus
FFQ: Food Frequency Questionnaire
USDA: United States Department of Agriculture
SD: Standard Deviation
BMI: Body Mass Index
PE: Preeclampsia
T2DM: Type 2 Diabetes Mellitus
CVD: Cardio Vascular Disease
MetS: Metabolic Syndrome
SFAs: Saturated Fatty Acids
MUFAs: Mono-unsaturated Fatty Acids
PUFAs: Poly Unsaturated Fatty Acids
OGTT: Oral Glucose Tolerance Test
IUGR: Intrauterine Growth Retardation
GPPAQ: General Practice Physical Activity Questionnaire
ANOVA: Analysis of Variance
ANCOVA: Analysis of Covariance
RRs: Relative Risks
CIs: Confidence Intervals
CRP: C-reactive Protein
TNF-a: Tumour Necrosis Factor-a
IL-6: Interleukin-6
IL-1β: Interleukin-1β
IL-4: Interleukin-4
IL-10: Interleukin-10
FFA: Free Fatty Acids
