# Supplementary material for: Association between dietary inflammatory potential and risk of developing gestational diabetes: a prospective cohort study
Source: Nutr J. 2021 Jun 2;20:48. doi: 10.1186/s12937-021-00705-5 (PMC8173880; doi:10.1186/s12937-021-00705-5)
Supplement: Supplementary file 1 — Additional file 1: Supplementary Table 1. Sample of the questionnaire. For each food item a portion size was considered. The frequency response options ranged from never to more than 6 times a day for each row. [file 12937_2021_705_MOESM1_ESM.docx]

**Supplementary table 1. Sample of the questionnaire. For each food item a portion size was considered. The frequency response options ranged from never to more than 6 times a day for each row**

| Food | Portion size | Never, or less than one per month | 1-3 per month | 1 per week | 2-4 per week | 5-6 per week | 1 per day | 2-3 per day | 4-5 per day | +6 per day |
| --- | --- | --- | --- | --- | --- | --- | --- | --- | --- | --- |
| Low fat milk | Cup |  |  |  |  |  |  |  |  |  |
| Trditional bread/Lavash | A loaf |  |  |  |  |  |  |  |  |  |
| Watermelon | A wedge |  |  |  |  |  |  |  |  |  |
| Cooked rice | Tbsp |  |  |  |  |  |  |  |  |  |
| Lentils | Cup |  |  |  |  |  |  |  |  |  |
| Yogurt drink (Dough) | Cup |  |  |  |  |  |  |  |  |  |
| Grapes | Cluster |  |  |  |  |  |  |  |  |  |
| Raisins | Tbsp |  |  |  |  |  |  |  |  |  |
| Mayonnaise | Tbsp |  |  |  |  |  |  |  |  |  |
| Lettuce | Cup |  |  |  |  |  |  |  |  |  |
